# Non-alcoholic fatty liver disease (NAFLD) and obesity in inflammatory bowel disease (IBD) patients in Gorgan

**DOI:** 10.22088/cjim.15.2.299

**Published:** 2024

**Authors:** Sima Besharat, Farideh Sakhavi, Parsa Sookhtehsaraei, Mehrdad Teimoorian, Somayeh Livani, Alireza Norouzi, Taghi Amiriani

**Affiliations:** 1 Infectious Diseases Research Center, Golestan University of Medical Sciences, Gorgan, Iran; 2 Golestan Research Center of Gastroentrology and Hepatology, Golestan University of Medical Sciences, Golestan, Gorgan, Iran; 3 Stem Cell Research Center, Golestan University of Medical Sciences, Gorgan, Iran; 4 Clinical Research Development Unit (CRDU), Sayad Shirazi Hospital, Golestan University of Medical Sciences, Gorgan, Iran

**Keywords:** Inflammatory bowel disease (IBD), Ulcerative colitis (UC), Crohn's disease (CD), Obesity, Non-alcoholic fatty liver disease (NAFLD)

## Abstract

**Background:**

According to the significance of extraintestinal symptoms in inflammatory bowel disease (IBD) patients and their connection with obesity, we aimed to investigate the prevalence of fatty liver in IBD patients of Sayyad Shirazi Hospital in Gorgan, Iran, in relation to obesity, anthropometric indicators and body image in these patients.

**Methods:**

Forty patients with IBD were recruited from all registered patients at the Golestan Research Center of Gastroenterology and Hepatology, following the specified inclusion and exclusion criteria. After obtaining written informed consent and filling in the questionnaire, the demographic and anthropometric indicators, and variables related to the disease were measured. The liver sonography was performed on all patients and graded by an expert radiologist. Data were analyzed using SPSS Version 16.0 statistical software at the significance level of 0.05.

**Results:**

We showed no significant difference between the distribution of demographic and anthropometric indicators in different groups of IBD patients. However, we demonstrated that the inappropriate values of HDL (0.004) and high values of LDL (0.015) were associated with fatty liver in IBD patients. Our findings also showed that NAFLD was significantly associated with overweight and obesity among IBD patients (P = 0.003).

**Conclusion:**

Our findings showed the epidemiological burden of NAFLD in IBD patients. Since fatty liver was associated with obesity, it is recommended that IBD patients be screened for risk factors associated with NAFLD to prevent liver disease.

*Inflammatory bowel disease* (IBD) is a chronic immune-related disorder in the intestine, which is divided into two main types *Ulcerative Colitis* (UC) and *Crohn's Disease* (CD) (1, 2). Currently, it is one of the common causes of gastrointestinal disorders in developing countries, including Iran (3, 4). Although the pathophysiology is not fully elucidated, the genetic background, immune system disorders, intestinal factors, and environmental elements affecting intestinal epithelial barriers and mucosal immune system could have significant roles in the pathogenesis of IBD (5-7). 

Although IBD-related mortality is not common, it causes disability and morbidity, especially in young adults, resulting in excessive social and economic burdens (8). The incidence rate of the UC and CD are 2-11 and 1-7 in 100,000, respectively (9, 10). The incidence of CD increased in developed countries from the middle of the 1950s to the beginning of the 1970s and has remained stable since then (11). The incidence of CD has been growing in Asian countries during recent years (12). 

Although the epidemiology of IBD is not well studied in Iran, one could say that IBD was a rare disorder in Iranian populations until 1985 and has started to rise since then (3). A large number of patients experience extraintestinal symptoms, including hepatobiliary disorders as the most common extraintestinal manifestation of IBD and NAFLD as the most important complication (13, 14). Considering that both intestinal inflammation and metabolic factors are effective in the pathogenesis of IBD-related NAFLD, a study showed that the risk factors for NAFLD in the IBD population are not different from those in the general population (15). Few studies on NAFLD screening in Asian patients with IBD have been done as well as a study in Taiwan that reported lower prevalence of NAFLD in IBD patients than in the western population. Therefore, it seems that the prevalence of NAFLD among patients with IBD is different based on the geographical region, time period of the study, environmental and genetic factors (16).

Obesity is a chronic medical condition that is defined as an excessive accumulation of body fat (17, 18). The global epidemic of overweight and obesity is rapidly becoming a major public health problem, leading to increased morbidity and mortality (19, 20). Obesity increases the risk of various diseases, especially heart disease, type 2 diabetes, obstructive sleep apnea, certain types of cancer, osteoarthritis, and asthma (21, 22). Several studies highlighted a possible biological relationship between obesity and IBD (23, 24). The mechanism by which obesity affects IBD is an area of active research (24). Since IBD patients have a higher volume of visceral adipose tissue, as the metabolically active fraction of body fat which may produce higher levels of pro-inflammatory cytokines, it may deteriorate disease progression and response to treatment in IBD patients (25, 26). Environmental factors affecting the occurrence of IBD include smoking, the level of health improvement and dietary changes related to lifestyle, and changes in the intestinal microbiome, including increased consumption of linoleic acid, increased consumption of fat/ animal protein, and fiber reduction in the diet (27, 28). Nowadays, one of the concerns of people is related to the appearance and shape of their bodies (29). Although *body dysmorphic disorder* (BDD) is considered an obsessive-compulsive disorder, it is still considered a body image disorder with socio-psychological and possibly biological effects, which might be exacerbated during obesity (30). 

The current study aimed to investigate the prevalence of fatty liver in IBD patients referred to the Gastroenterology Clinic of Sayyad Shirazi Hospital in Gorgan, Iran, in relation to obesity and the determination of anthropometric indicators and body image in these patients. 

## Methods

**Ethical Considerations** ***:*** The study procedure was reviewed and approved by the Ethics Committee of the Golestan University of Medical Sciences (GoUMS) (Code of Ethics: IR.GOUMS.REC.1399.101). A written informed consent was obtained from all participants after explaining the study procedure. It was clearly explained to all participants that all possible complications should be informed and the patients were referred to the specialist as soon as possible. 

**Patients and study procedure:** All patients with IBD who were registered at the registry center of Golestan Research Center of Gastroenterology and Hepatology were invited to participate in this cross-sectional study in 2020, and 40 patients were recruited according to the specified criteria. The power of the study was considered to be 95%, and the α error was 0.05. 

**The inclusion criteria:** The inclusion criteria comprised of having the intestinal disease for at least two years and not having a history of taking antihyperlipidemic drugs. After obtaining written informed consent and filling in the questionnaire, the demographic and anthropometric indicators, including height, weight, gender, smoking, concomitant diseases (diabetes, high blood pressure, high blood fat), and variables related to the disease including duration of IBD with confirmed pathology report, new IBD (UC and CD), waist circumference and abdominal circumference of the patients were measured. 

**The exclusion criteria:** All of the patients who were in the acute phase of confirmed IBD, hospitalized, or had a history of cardiac surgery or consuming anti-hyperlipidemic drugs/ immunosuppressants/ corticosteroids in the past six months were excluded. 

**Measurements:** The liver sonography was performed on all patients using a UGEO WS80A ultrasound system (Samsung Medison, Seoul, Korea). A radiologist at that hospital graded the condition of the liver based on the existing criteria as follows, 1- Liver with normal echogenicity, 2- The grade 1 fatty liver in which the echogenicity of the liver is found, 3- The grade 2 fatty livers in which the echogenicity of the liver caused the walls of the portal vein branches to become unclear, 4- The grade 3 of fatty liver in which the limits of the diaphragm are unclear (31). By examining the sonography results of the patients, those with fatty liver (with any grade) were referred for further investigations and necessary treatments. The abdominal circumference of patients was measured and divided into two groups of proportionate and disproportionate (Above 102 in men and above 88 in women). Lipid profile and liver function tests were analyzed in the clinical laboratory of Seyyed Shirazi Hospital and HDL levels were divided into two categories of appropriate (over 60 mg/dL) and inappropriate (lower than 60 mg/dL), while LDL levels were divided into two categories of appropriate (lower than 100 mg/dL) and inappropriate (higher than 100 mg/dL).

**Statistical Analysis:** Data were analyzed using SPSS Version 16.0 statistical software. The normality of the data was first assessed using the Kolmogorov-Smirnov test. The parametric independent samples t-test and one-way ANOVA, or the nonparametric Mann-Whitney and Kruskal-Wallis tests, were used to compare the means between groups. In the analysis of qualitative data, chi-square or Fisher's exact tests were used. The significance level in all tests was considered less than 0.05.

## Results

**Demographic characteristics and the analysis of BMI, abdominal circumference and body image:** All patients with IBD who were registered in the registration system of Golestan Research Center of Gastroenterology and Hepatology were invited to participate in this study and 40 patients were recruited (of which the body image was assessed in 34 patients), according to the inclusion and exclusion criteria. Individual characteristics of patients with IBD are listed in table 1. The quantitative characteristics are demonstrated in means ± S.D, while the qualitative characteristics are shown in number and percent. As demonstrated in table 2, we demonstrated no significant difference between the distribution of BMI (P = 0.754), abdominal circumference (P = 0.329) and body image (P = 0.581) in two gender groups (male and female) of IBD patients (table 2). 

**Figure1:**
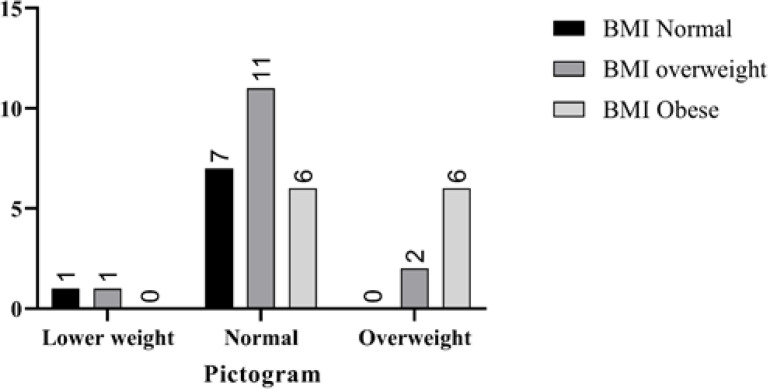
Comparison of the BMI of the examined patients with the body image, We compared the BMI of all patients with their body image scores and showed that the body image scores are different from the actual size, while some overweight or obese patients assume themselves in the normal range

We also compared the BMI of all patients with their body image scores and showed that the body image scores are different from the actual size, while some overweight or obese patients assume themselves in the normal range (fig1).

**Fatty liver in overweight and obese IBD patients:** As demonstrated in table 2, sonography examinations revealed the evidence in favor of fatty liver presence among 17 (7 males and 10 females) patients, which was not statistically significant (P = 0.50). Among these 17 patients, 15 patients were diagnosed with UC and two patients demonstrated CD, which was not also statistically significant (P = 0.10). Furthermore, all 17 patients were overweight or obese, which resulted in a significant difference between two groups (P = 0.003) (table 2).

**Lipoprotein profile in IBD patients:** We compared the laboratory findings and sonography reports of the IBD patients. As shown in table 3, the inappropriate values of HDL (0.004) and high values of LDL (0.015) were associated with fatty liver in IBD patients (table 3). 

**Table 1:** Individual characteristics of patients with inflammatory bowel disease

**Characteristic**		**Mean/Number**	**S.D/Percent**
**Age (year)**		44.30	9.76
**Duration of IBD (year)**		9.47	6.44
BMI*		27.82	4.71
**BMI (qualitative assessment)**	**Normal**	8	23.5 %
	**Overweight**	14	41.2 %
	**Obese**	12	35.3 %
**Gender**	***Male***	14	35 %
	***Female***	26	65 %
**Type of IBD**	***Ulcerative colitis***	34	85 %
	***Crohn’s disease***	6	15 %
**Involved location**	**Left colon**	22	55 %
	**Pancolitis**	12	30 %
	**Other locations**	6	15 %
**Fatty Liver**		17	42.5 %
**Fatty Liver grades**	**1**	15	88.2 %
	**2**	2	11.8 %
**Abdominal circumference (cm)**	**Normal**	16	47.1 %
	**Above 102 in men and above 88 in women**	18	52.9 %
**Body Image**	**Lower weight**	2	5.9 %
	**Normal**	24	70.6 %
	**Overweight**	8	23.5 %

BMI: body mass index, 34 patients were successfully analyzed for BMI, body image and abdominal circumference.

**Table 2:** Distribution of BMI, waist circumference, body image and fatty liver in patients with IBD according to gender

**Characteristic**		**Gender**		***P*** **-value**
		**Male** **N (%)**	**Female** **N (%)**	
**BMI**	**Normal**	2 (20)	6 (25)	0.754
	**Overweight and Obese**	8 (80)	18 (75)	
**Abdominal circumference (cm)**	**Normal**	6 (60)	10 (41.7)	0.329
	**Above 102 in men and above 88 in women**	4 (40)	14 (53.8)	
**Body Image**	**Normal**	7 (70)	19 (79.1)	0.581
	**Overweight**	3 (30)	5 (20.8)	
**Fatty Liver in Sonography**		7 (41.2)	10 (58.8)	0.50

**Table 3:** Investigating the distribution of fatty liver according to laboratory measures in patients with IBD

**Laboratory measures**		**Diagnosed with Fatty Liver**		***P*** **-value**
		**No** **N (%)**	**Yes** **N (%)**	
**Total Cholesterol (mg/dl)**	**≤ 200**	18 (78.3)	12 (70.6)	0.60
	**> 200**	5 (21.7)	5 (29.4)	
HDL (mg/dL) *	**> 60 (Appropriate)**	13 (56.5)	4 (23.5)	**0.004**
	**< 60 (Inappropriate)**	10 (43.5)	13 (76.5)	
**LDL (mg/dL)**	**≤ 100**	17 (74)	6 (35.3)	**0.015**
	**> 100**	6 (26)	11 (64.7)	
AST (U/L) *		30.18 ± 15	34.32 ± 16	0.411
**ALT (U/L)**		24.7 ± 24.8	28.73 ± 25.34	0.699
**ALP (U/L)**		177/7 ± 199.11	131 ± 23.21	0.409

Means ± S.D. HDL: high density lipoprotein, LDL: low density lipoprotein, AST: aspartate transaminase, ALT: alanine transaminase, ALP: alkaline phosphatase.

## Discussion

Non-alcoholic fatty liver disease (NAFLD) has become one of the most critical diseases of the liver and an important health issue with growing morbidities (32). Despite recent scientific achievements, the etiology of NAFLD still has remained unknown and no effective medication is available to prevent its progression (33). As a manifestation of systemic metabolic syndrome, NAFLD is closely related to glucose and lipid metabolism and is often observed in obese populations (34, 35). However, many non-obese people are diagnosed with NAFLD during clinical examinations, and the mechanism of NAFLD occurrence in non-obese people is under more debates (36).

Here, we diagnosed NAFLD in 42.5% of IBD patients by imaging, which was higher than the reported prevalence in the general population of the United States (approximately 33.6% of IBD patients) (37). However, other studies reported lower frequencies of NAFLD (approximately 10% of IBD patients) (38, 39). On the other hand, the frequency of IBD patients suffering from metabolic syndrome or susceptible to was 51.5% in our study, which was similar to the reports from the general population of the United States (40, 41). In a recent cohort study from Greece, NAFLD was reported as CD in 14.2% and UC in 26.2% of IBD patients (4), which were lower frequencies in comparison to our findings (CD in 33.33% and UC in 44.11% of IBD patients). The reports from Bargiggia et al. (2016) (42) and Hoffmann et al. (2003) (43) were controversial and different from our findings, which could be justified by different eating habits in the studied regions. Another important factor that should be considered is the improvement of IBD treatments and, as a result, better management of malnutrition in IBD patients over time. In a meta-analysis by Vaisi-Raygani et al., the average frequency of overweight individuals in the general Iranian population of adults was 21.4% (44), which was markedly lower than the frequency in our study (76.5%). Accordingly, obesity, metabolic syndrome and being overweight could be considered as the main factors involved in increasing the susceptibility of NAFLD, which should be confirmed in further investigations. 

Dyslipidemia (reduction of HDL and elevation of LDL) was observed with a significant statistical difference in our study, delineating the companionship between IBD and NAFLD. Other studies in different regions demonstrated controversial results, of which Hoffmann et al. (43) was in accordance with our findings, while some other reports were incongruent (45). The demonstrated controversies could be due to the different nutritional pattern in each geographic region, the sample size differences, the utilized laboratory methods, and the demographic discrepancies of the studied populations. There were several limitations to our study including the lack of access to transient elastography (TE) with vibration control or MR spectroscopy for grading steatosis and fibrosis. Liver histology was not investigated and since the comparison between NAFLD and NASH is only possible with histology, there could be an evaluation error. Moreover, there was no reliable information about the nutrition or exact metabolic status of the patients. Accordingly, it is suggested to investigate more variables in larger population, preferably during a prospective cohort. Our findings showed the epidemiological burden of NAFLD in IBD patients. Based on the results of our study, fatty liver was significantly associated with overweight and obesity among IBD patients. The laboratory findings indicated an impaired lipid profile in IBD patients with fatty liver. Based on our results, it is suggested to screen IBD patients for risk factors associated with NAFLD to prevent liver disease. Moreover, the timely diagnosis of fatty liver by reliable methods could and modifying dietary habits could be suggested to improve the metabolic profile of IBD patients. Furthermore, our findings showed that IBD care should not be limited to intestinal disease, but should include metabolic interventions by promoting a healthy lifestyle and a proper diet to reduce the emergence of liver disease.
